# Physics-guided machine learning for real-time, non-contact quantification of liquid volume at micro litter under cyclone flow

**DOI:** 10.1007/s44211-026-00869-2

**Published:** 2026-01-22

**Authors:** Chenyu Zhou, Ruying Wang, Sangming Xu, Roichi Ohta, Hidekatsu Tazawa, Kazuma Mawatari

**Affiliations:** Graduate School of Information, Production and Systems, Waseda University, 2-7 Hibikino, Wakamatsu-ku, Kitakyushu, Fukuoka, 808-0135 Japan

## Abstract

Cyclone flow is a technique to evaporate the liquid at room temperature. Efficient milliliter-to-microliter sample concentration at room temperature is expected for high-sensitivity analysis in biology and chemistry; however, it is hindered by the difficulty of quantifying the volume in real-time under the cyclone flow. This research targets a non-contact, vision-based system using a physics-guided machine learning (PGML) framework to precisely monitor and control this process under intense cyclone flow. The key is a physics-informed loss function that embeds the container’s geometric constraints into the neural network’s training, substantially enhancing model robustness and accuracy. Experimental results demonstrate the PGML model’s superiority, achieving a nearly 70% reduction in error compared to purely data-driven methods. The system shows a measurement error of just 1.2% and a coefficient of variation of 1.5% at a 20 µL target, meeting stringent bioassay requirements. This work establishes a powerful solution for automated and precisely quantitative sample concentration, promising to advance a wide range of analytical applications.

**Graphical abstract:**

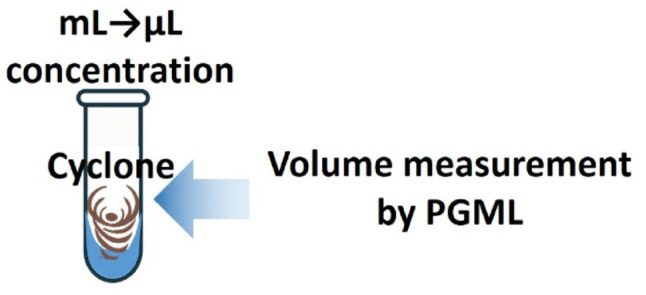

**Supplementary Information:**

The online version contains supplementary material available at 10.1007/s44211-026-00869-2.

## Introduction

Highly sensitive and quantitative analysis stands as a foundational pillar of modern science, enabling critical advancements in fields from clinical medicine and life sciences to environmental protection and food safety. Its importance is particularly pronounced in biomedical applications, where the ability to detect trace amounts of biomarkers—such as circulating tumor DNA (ctDNA), circulating tumor cells (CTCs), and exosomes—is paramount for realizing “liquid biopsies” [1]. The precise quantification of these entities, often present at nanogram or even picogram per milliliter levels, is the key to early disease diagnosis, post-treatment monitoring, and the overall advancement of precision medicine [2–4]. However, the concentration of these critical biomarkers in raw biological samples frequently falls below the inherent limit of detection (LOD) of even the most powerful analytical instruments, creating a significant sensitivity gap [5, 6]. To bridge this gap, an efficient and lossless pre-concentration step to enrich the target analyte is indispensable [7].

To address this need, various sample concentration strategies have been developed, yet they face significant trade-offs that compromise sample integrity. Techniques such as heat-based evaporation, while effective at solvent removal, risk the irreversible thermal denaturation of fragile macromolecules like proteins and nucleic acids, catastrophically destroying their bioactivity [8]. Other common methods, including ultrafiltration and solid-phase extraction, suffer from significant and often unpredictable analyte loss due to non-specific adsorption to membranes or media [9, 10]. This issue is particularly acute for low-abundance samples, where poor and inconsistent recovery rates can severely undermine quantitative accuracy, leading to analytical failure or false-negative results. Consequently, there remains an urgent need for a concentration paradigm that is simultaneously gentle, efficient, and ensures high, reproducible recovery.

A highly promising alternative is room-temperature cyclone-based concentration, which utilizes principles of fluid dynamics to accelerate solvent evaporation without resorting to damaging heat or physical contact with membranes [11]. By generating a high-speed vortex in the headspace above a sample, this technology dramatically increases the gas-liquid interface area, enabling rapid, efficient concentration while preserving the analyte’s native structure and function. However, the critical challenge of this technique lies in quantification. To address this, hardware-based solutions have been developed, such as devices with integrated microchannels for endpoint volume measurement after concentration is complete [12]. While effective for final quantification, these methods lack the capability for real-time monitoring and are inflexible for stopping at arbitrary volumes. The core problem remains: the intense vortex flow make precise, real-time volume measurement—a prerequisite for automated process control—difficult with conventional methods. This inability to accurately and dynamically quantify the process has severely restricted the practical application of this technology in quantitative analytical fields.

As mentioned above, quantifying the volume from images under the cyclone flow is a highly complex visual recognition task that is difficult to solve with traditional algorithms. However, recent advancements in machine learning, particularly convolutional neural networks (CNNs), offer a powerful data-driven approach to interpreting complex visual information [13]. In theory, a CNN could learn direct mapping from an image of a dynamic vortex to its corresponding volume. However, these “black-box” models are heavily dependent on vast and comprehensive training datasets and are prone to poor generalization when faced with unforeseen conditions [14]. More critically, they lack any inherent understanding of physics and can produce physically implausible predictions, a fatal flaw for scientific measurement applications where reliability is paramount. Specifically for the cyclone flow case, the violent and fluctuating liquid surface creates a visually complex environment with transient ripples, reflections, and an unstable vortex shape. A pure ML model is susceptible to learning fake correlations from these visual artifacts, mistaking them for indicators of volume. Consequently, its performance degrades sharply when faced with slight variations in flow rate or lighting that alter these superficial features. Physics-Guided Machine Learning (PGML) addresses this limitation directly [15]. By embedding the container’s known geometric relationship between liquid height and volume into the training process, we impose a hard physical constraint on the model. This constraint acts as a powerful regularizer, forcing the network to disregard the misleading visual noise of the vortex and instead learn the more fundamental, physically-grounded features that determine the true volume. This results in a model that is not only more accurate but also significantly more robust and generalizable to the dynamic conditions of the cyclone flow.

We have developed and validated a low-cost, non-contact measurement system that integrates a vision-based deep learning model with fundamental physical principles. The core of our technique is a custom, physics-informed loss function that mathematically encodes the known geometric constraints of the sample container directly into the model’s training process. This hybrid approach compels the model to learn a physically self-consistent and therefore more robust relationship between image features and liquid volume. This study demonstrates that our PGML-based system can accurately and reliably quantify microliter-level volumes in real-time, even under the most intense cyclone flow, finally bridging the critical quantification gap and unlocking the full potential of this advanced concentration technology.

## Experimental methods

**Fig. 1:**
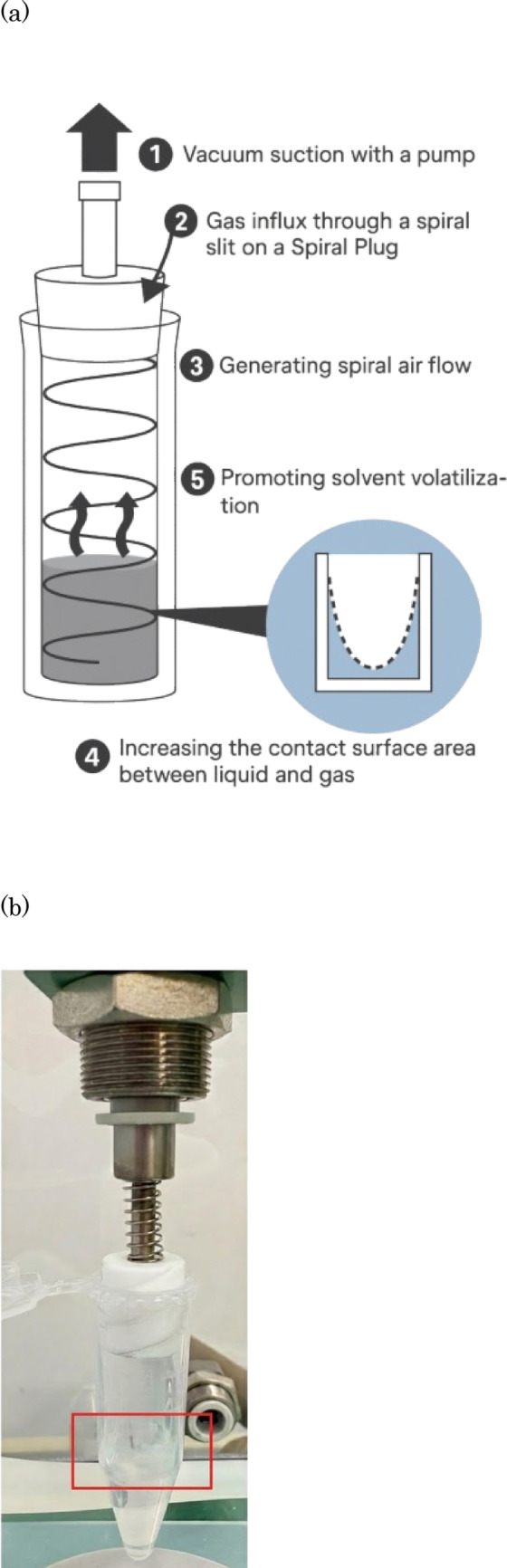
Explanation of cyclone-based concentration method: **a** the working principle of cyclone-based concentration and** b** a practical example of the device.

**Fig. 2:**
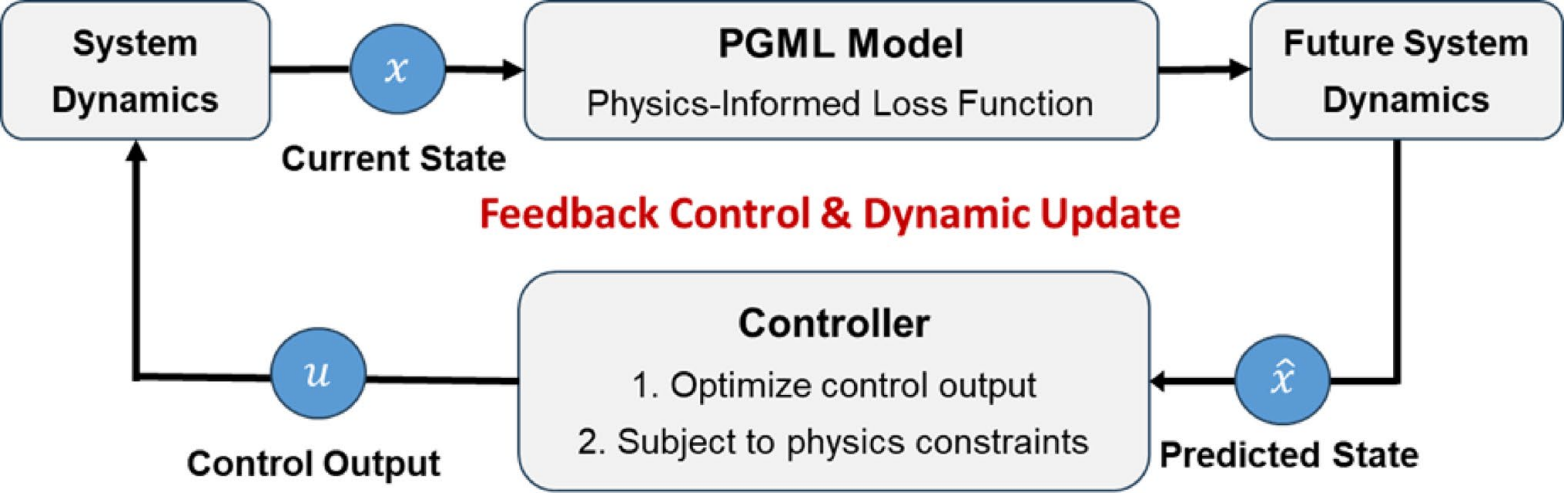
PGML-guided automatic concentration process showing the feedback control loop

**Fig. 3:**
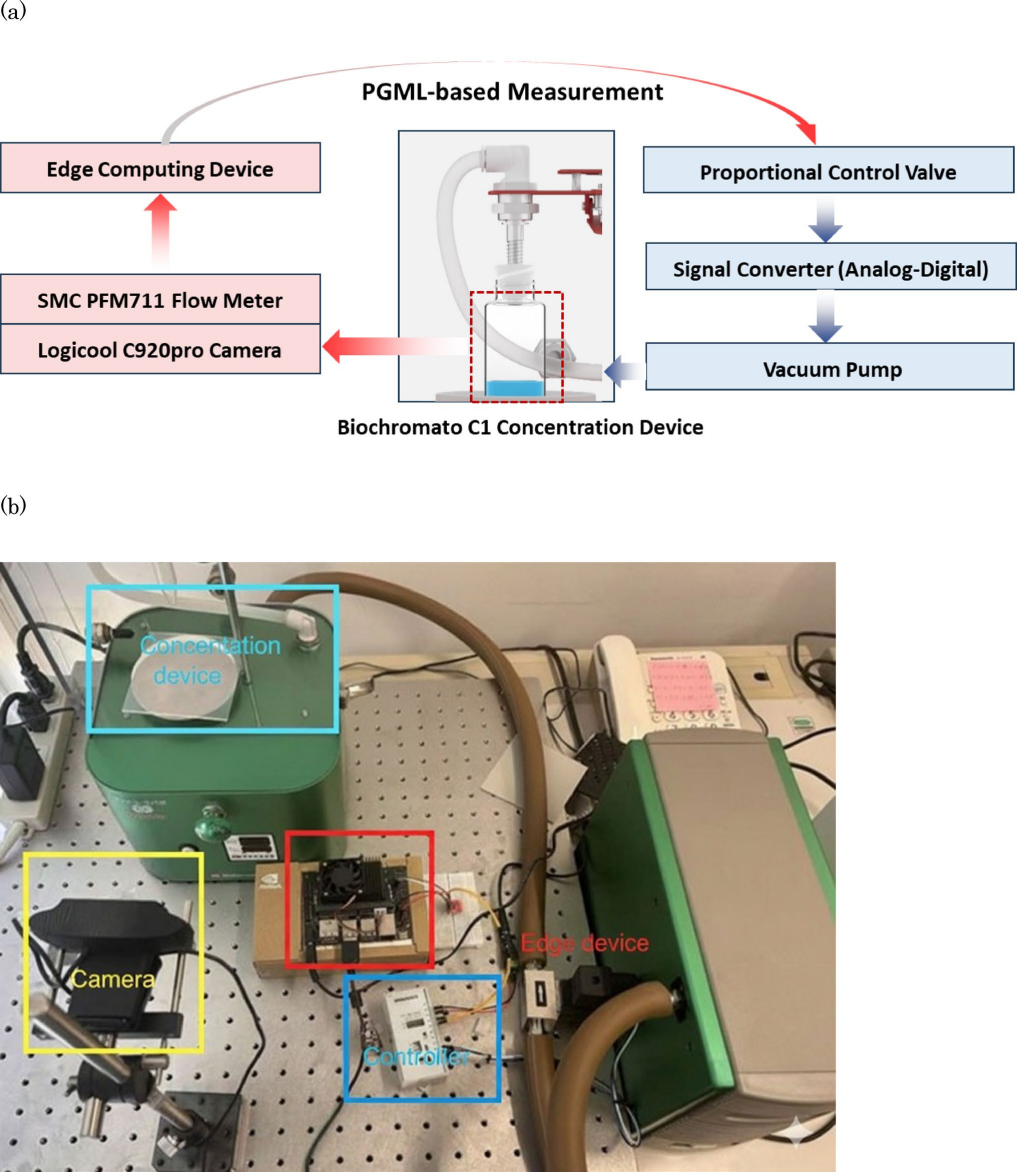
Experimental system:** a** the overall experimental system design, including the concentration device, camera, and edge computing unit and** b** a photo of the system

A low-cost, non-contact measurement system was designed to achieve real-time quantification through an automated feedback control loop, as conceptualized in Fig. 1. The model’s real-time predictions guide the system’s physical state to achieve a precise target volume. The hardware consists of three main modules: (1) a sample processing module (Model C1 cyclone concentrator, BioChromato, Inc.) to generate a stable vortex in a standard 5mL Eppendorf tube [16]; (2) an image acquisition module using a Logicool C920 Pro HD Webcam to capture a 1080p video stream of the sample; and (3) an edge computing and control module (NVIDIA Jetson Nano Developer Kit), which processes the visual data and actuates a proportional control valve to manage the gas flow. The software, developed in Python with OpenCV and PyTorch, captures and preprocesses images, feeds them to the measurement model for inference, and uses the output to enable the closed-loop control, as Fig. 2 shows. The entire system design is shown in Fig. 3.

The core of our approach is a measurement model that integrates physical principles into a deep learning framework. The 5mL Eppendorf tube is modeled as a composite shape of a cylinder and a cone. The relationship between liquid volume $\:V$ and liquid height $\:h$ (measured from the cone’s vertex) is described by a precise, piecewise function $\:V=f\left(h\right)$, representing a priori physical knowledge for our model. For $\:\left(0\le\:h\le\:{H}_{2}\right)$, this function is defined as

1 $$V=\frac{1}{3}\pi {\left( {\frac{{{r_2}}}{{{H_2}}}h} \right)^2}h=\frac{{\pi r_{2}^{2}}}{{3H_{2}^{2}}}{h^3}$$.

When the liquid level is in the cylindrical section $\:\left({H}_{2}\le\:h\le\:{H}_{1}+{H}_{2}\right)$,

2 $$V={V_{con{e_f}ull}}+{V_{cylinder}}=\frac{1}{3}\pi r_{2}^{2}{H_2}+\pi r_{1}^{2}\left( {h - {H_2}} \right)$$,

where $\:{r}_{1}$ and $\:{H}_{1}$ are the radius and height of the cylinder, and $\:{r}_{2}$ and $\:{H}_{2}$ are the radius and height of the cone. To embed this geometric constraint, we designed a neural network with two outputs: predicted volume $\:\widehat{V}$ and predicted height $\:\widehat{h}$. The physics-informed loss term, $\:{L}_{physics}$, penalizes any violation of this relationship. It is defined as the mean squared error of a physics residual $\:{P}_{\theta\:}$:

3 $${P_\theta }=\hat {V} - f\left( {\hat {h}} \right)$$

Therefore, the physics-informed loss term above, $\:{L}_{physics}$​, is defined as the MSE of this physics residual over a batch of M samples:

4 $${L_{physics}}=\frac{1}{M}\sum\limits_{{j=1}}^{M} {{{\left( {{{\hat {V}}_j} - f\left( {{{\hat {h}}_j}} \right)} \right)}^2}=} \frac{1}{M}\sum\limits_{{j=1}}^{M} {{{\left( {{P_{\theta ,j}}} \right)}^2}}$$

This term enforces physical self-consistency without requiring ground-truth labels. The model is trained by minimizing a total loss function that combines the physics-informed loss with a standard supervised data loss, $\:{L}_{\mathrm{d}\mathrm{a}\mathrm{t}\mathrm{a}}$. The data loss measures the weighted mean squared error between the model’s predictions $\:\left(\widehat{V},\widehat{h}\right)$ and the ground-truth labels $\:\left(V,h\right)$:

5 $${L_{data}}={L_v}+\alpha {L_h}=\frac{1}{N}\sum\limits_{{i=1}}^{N} {{{\left( {{{\hat {V}}_i} - {V_i}} \right)}^2}} +\alpha \frac{1}{N}\sum\limits_{{i=1}}^{N} {{{\left( {{{\hat {h}}_i} - {h_i}} \right)}^2}}$$

Where $\:\alpha\:$ is a weighting coefficient used to balance the importance of the two tasks. The total loss is a weighted sum:

6 $${L_{total}}={\lambda _{data}}{L_{data}}+{\lambda _{physics}}{L_{physics}}$$

The weighting coefficients $\:{\lambda\:}_{data}$ and $\:{\lambda\:}_{physics}$ govern the critical trade-off between minimizing the data-driven prediction error and satisfying the underlying physical laws. In this study, these hyperparameters were determined empirically through a comprehensive grid search on the validation set. Our analysis revealed that assigning a dominant weight to the physics-informed loss ($\:{\lambda\:}_{physics}=10$) was indispensable for guiding the optimization process toward a physically plausible solution space. This strong regularization compels the network to prioritize the container’s geometric constraints over transient visual noise, such as rapid surface fluctuations and complex optical reflections. Consequently, this configuration prevents the model from learning spurious correlations in the early stages of training and ensures robust generalization under dynamic flow conditions.

**Fig. 4:**
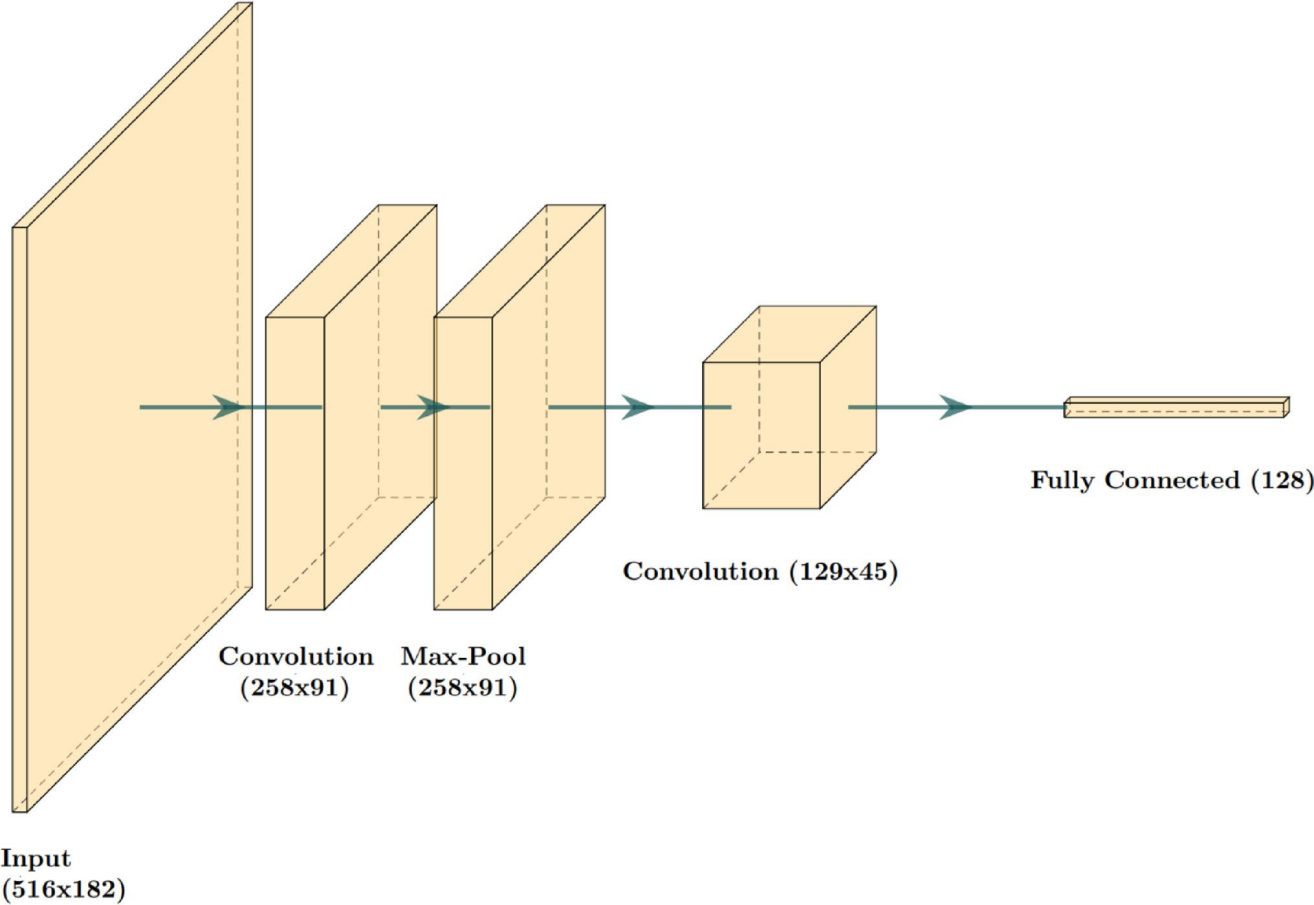
Schematic diagram of the proposed Convolutional Neural Network (CNN) architecture

A lightweight CNN was designed for efficient inference on the edge device. The architecture consists of two convolutional blocks for feature extraction, followed by a global average pooling layer and a fully connected output layer that regresses to the volume and height values, as shown in Fig. 4.

For experimental validation, a dataset of 7493 images was collected, capturing deionized water volumes from 20 to 50 µL under cyclone flow rates varying from 0 L/min (static) to 15 L/min (high intensity). The dataset was randomly split into training (80%), validation (10%), and testing (10%) sets. Model performance was evaluated using two primary metrics: the Average Percentage Difference (APD) for accuracy and the Coefficient of Variation (CV) for precision and stability. As for the performance metrics employed for evaluation, APD was utilized to assess model accuracy, defined as the mean of absolute percentage errors between the predicted and true values. As a common metric in machine learning for regression tasks, APD provides an intuitive summary of the overall error magnitude. The CV served as the key indicator for precision and repeatability. Defined as the ratio of the standard deviation to the mean for a set of measurements, a low CV signifies exceptional system stability and consistency.

## Results and discussion

The primary challenge of this research is to achieve accurate, real-time volume quantification under the turbulent conditions created by intense cyclone flow. To validate our proposed PGML approach, we first benchmarked its performance against a purely data-driven ML model under a high-intensity and varying flow rate in Video S1. The results conclusively demonstrated the transformative advantage of integrating physical constraints. The pure ML model’s performance degraded sharply in the presence of a vortex, with its APD reaching a high value. This is because a purely data-driven model is susceptible to learning spurious correlations from transient visual artifacts, such as surface ripples or reflections, which are abundant in a dynamic vortex.

In contrast, the PGML model maintained exceptional accuracy and stability across all tested conditions. By embedding the container’s geometric relationship between volume and height as a physics-informed loss function, the PGML model is compelled to learn a more robust and generalizable representation. This physics-informed regularization effectively allows the model to overlook the visual noise of the vortex and infer the true volume based on global morphology, thus verifying that our principle is sound and effective for this complex application.

**Fig. 5:**
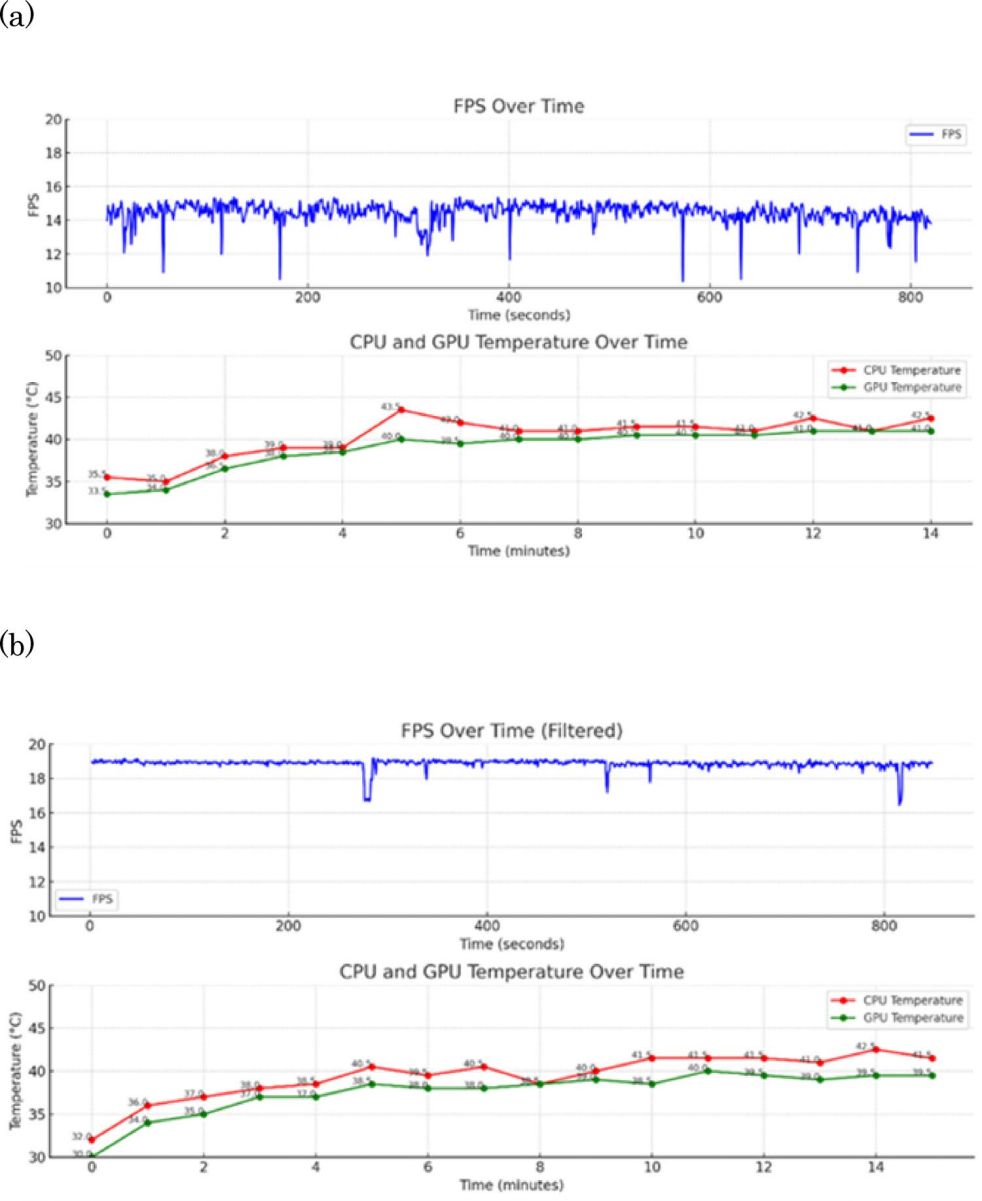
Real-time inference performance on the edge device, comparing Frames Per Second (FPS):** a** before TensorRT optimization and** b** after TensorRT optimization.

Next, for the system to be practically viable in a laboratory setting, real-time inference and closed-loop control are essential. Therefore, we optimized the trained PyTorch model for deployment on the NVIDIA Jetson Nano edge computing device. We utilized NVIDIA’s TensorRT engine with INT8 quantization to accelerate inference. This optimization yielded a substantial performance increase, boosting the average processing speed from an unstable ~ 12 FPS to a stable ~ 19 FPS. As shown in Fig. 5, this throughput is more than sufficient for real-time monitoring and feedback control, all while maintaining safe operational temperatures on the device without the need for additional cooling hardware.

With the principle validated and the model optimized, we conducted a comprehensive evaluation of the final system’s accuracy and precision. The PGML model’s performance was compared to the baseline ML model under both static (0 L/min) and dynamic (15 L/min) conditions, with the results summarized in Tables 1 and 2.

**Table 1:** Model performance comparison under static conditions (0 L/min)

Volume (µL)	APD (PGML) (%)	CV (PGML) (%)	APD (ML) (%)	CV (ML) (%)
50	0.22	0.35	0.70	0.75
40	0.40	0.42	0.80	0.88
30	0.37	0.48	0.87	0.92
20	0.45	0.50	0.90	0.95

**Table 2:** Model performance comparison under cyclone flow (15 L/min)

Volume (µL)	APD (PGML) (%)	CV (PGML) (%)	APD (ML) (%)	CV (ML) (%)
50	1.12	0.96	3.84	2.85
40	1.35	1.10	4.57	3.58
30	1.78	1.42	6.32	4.71
20	2.43	2.10	8.96	6.95

Under static conditions, both models performed well. However, the PGML model consistently showed lower APD and CV, suggesting that the physics constraint acts as an effective regularizer even in the absence of a vortex. Under dynamic cyclone flow, the superiority of the PGML approach became overwhelmingly clear. As detailed in Table 2, the PGML model reduced the APD by nearly 70% on average compared to the pure ML model. Even at the most challenging low-volume measurement point of 20 µL, our system achieved an outstanding APD of just 2.43% and a CV of 2.10%. This level of precision is within the 2–5% threshold typically considered acceptable for analytical chemistry, validating its suitability for demanding applications like high-precision bioassays.

**Fig. 6:**
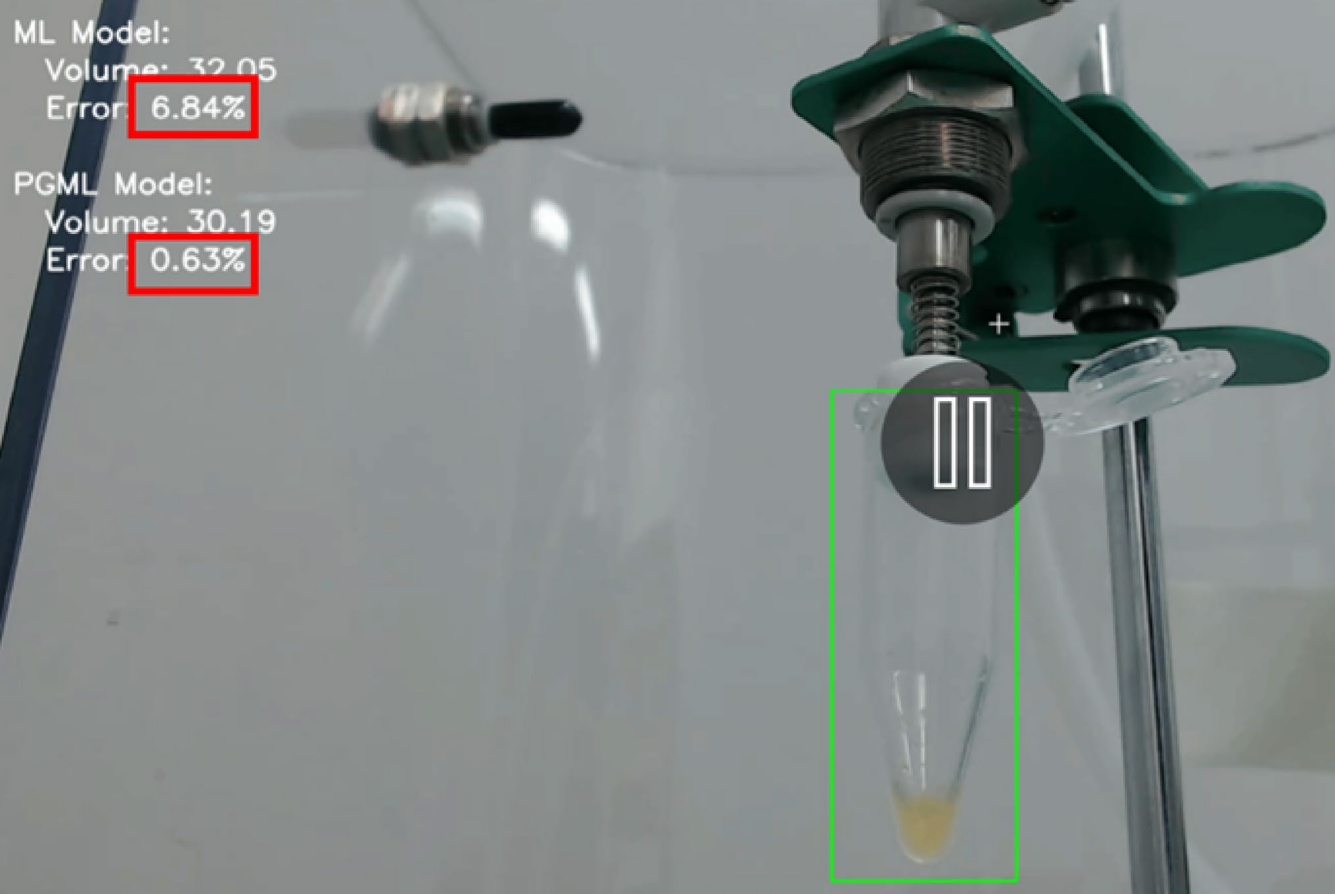
Screenshot of the real-time application comparing the predictions of the pure ML and PGML models on a dynamic liquid sample.

A live demonstration from a screenshot of Video S1 (Fig. 6) further illustrates this practical impact. The exceptional performance demonstrated above by our system is fundamentally attributed to the innovative application of the PGML framework. Traditional, purely data-driven models, when faced with the violent and irregular liquid surface created by the cyclone flow, are highly susceptible to interference from transient visual noise such as surface ripples and reflections. This leads them to learn spurious correlations, which in turn degrades prediction accuracy and stability. In contrast, our PGML model, by encoding the container’s geometric and physical constraints into its loss function, effectively compels the neural network to disregard these superficial and irrelevant visual artifacts. The model learns to look beyond the ever-changing vortex shape and instead focuses on the more fundamental, stable relationship between the liquid’s macroscopic morphology and its volume, as dictated by physical laws. It is precisely this inherent robustness against dynamic visual interference that forms the core reason our system can achieve high-accuracy and high-repeatability measurements.

While our system represents a significant advance, it is important to acknowledge the limitations of the current study. The exceptional results were achieved within a specific, controlled experimental context, as the model was trained specifically using a dye solution. Therefore, its generalizability to substantially different scenarios—such as solutions with varying colors and transparencies or novel container types—requires further validation and would likely necessitate additional computer vision processing or model retraining.

Nevertheless, the foundational design of the PGML model offers a significant advantage in robustness over traditional machine learning methods. By anchoring its predictions to the invariant physical constraints of the container, the model is intrinsically less dependent on superficial visual features. This suggests that even when faced with the visual noise of a new environment, its focus on geometric principles would confer a higher degree of stability, representing a distinct advantage over conventional models that are more prone to performance degradation.

Future investigations should focus on creating a more generalized model, potentially by incorporating container and liquid properties as inputs. Additionally, a valuable next step would be to implement more sophisticated control algorithms, such as PID (Proportional-Integral-Derivative) control, to dynamically regulate the concentration rate and further enhance the system’s utility and automation capabilities. Consequently, this work not only provides a solution for a specific application but also offers a guiding principle: integrating domain-specific physical laws is a critical strategy for building robust machine learning systems for complex scientific measurement, paving the way for future advancements in similar applications.

While this study focuses on the high-precision geometric quantification of liquid volume, we acknowledge the importance of validating the chemical enrichment factor. Given the low volumetric error achieved (APD < 2.5%), the system theoretically ensures accurate concentration ratios. Future work will also explicitly correlate these volumetric measurements with analytical recovery rates determined by methods such as HPLC (high performance liquid chromatography) or CE (capillary electrophoresis) to further validate the system’s applicability in complex bioassays.

## Conclusion

In this work, we have successfully developed and validated a non-contact, real-time measurement system that resolves the critical quantification challenge in room-temperature cyclone concentration. By pioneering the use of a Physics-Guided Machine Learning (PGML) model, we have demonstrated that embedding geometric constraints into the neural network’s learning process enables highly accurate and robust volume measurements, even under the turbulent conditions of a dynamic vortex.

Our key contribution is a complete, low-cost system that significantly outperforms traditional data-driven models, achieving a precision level (CV < 2%) suitable for demanding analytical applications. This research not only transforms cyclone concentration into a viable and quantitative tool for lossless sample preparation in fields like biomedicine and diagnostics but also presents a powerful framework for developing intelligent, physics-aware measurement systems for other complex scientific challenges. This approach paves the way for a new generation of smart laboratory instruments that are more accurate, automated, and reliable.

## Supplementary Information

Below is the link to the electronic supplementary material.

Supplementary Material 1

## Data Availability

The key experiment parameters have been included in this manuscript and the dataset that support the findings of this study are available from the corresponding author upon reasonable request.
